# Effects of *Broussonetia papyrifera* silage on rumen fermentation parameters and microbes of Holstein heifers

**DOI:** 10.1186/s13568-022-01405-x

**Published:** 2022-05-25

**Authors:** Zhiying Wen, Yiye Chen, Longfei Wu, Hanchen Tian, Ni Zhu, Yongqing Guo, Ming Deng, Jianying Liu, Baoli Sun

**Affiliations:** 1 College of Animal Science, South China Agricultural University, Guangzhou, 510642 China; 2 Agro-Tech Extension Center of Guangdong Province, Guangzhou, 510520 China

**Keywords:** *Broussonetia papyrifera*, Holstein heifer, Rumen microbes, WGCNA, High-throughput sequencing

## Abstract

The structure and types of rumen microbes are closely related to host health. This study aimed to evaluate the effect of *Broussonetia papyrifera* silage (*BPS*) gradually replacing the whole crop maize silage (WCMS) on total tract digestibility, rumen fermentation parameters, serum biochemical indicators, and rumen microbes of Holstein heifers. The diet treatment consisted of four proportions of *BPS* (0%, 25%, 50% and 75%) as substitute for WCMS (designated as T0, T25, T50 and T75, respectively). Twenty heifers (body weight = 245 ± 24 kg) were randomly divided into four groups of five heifers, and randomly received one diet. The feeding adaption period was 7 days, and the experiment period was 30 days. Our findings suggested that the digestibility of neutral detergent fiber and crude protein increased linearly with the increased in *BPS* (*P* < 0.05). The concentrations of total protein and albumin increased quadratically with the increased in *BPS* (*P* < 0.05). The 16s high-throughput sequencing showed that feeding *BPS* did not change the diversity and structure of the rumen microbes of heifers. However, the relative abundances of *Tenericutes* and *SR1–Absconditabacteria* increased linearly with the increased in *BPS* (*P* < 0.05). The Weighted Correlation Network Analysis results suggested that ALT concentration was positively correlated with the abundance of *Prevotella-1* (r = 0.73; *P* = 0.007). In general, Holstein heifers fed with *BPS* did not change the diversity and uniformity of rumen microbes, and enhanced the body protein metabolism.

**Supplementary Information:**

The online version contains supplementary material available at 10.1186/s13568-022-01405-x.

## Introduction

Maize (*Zea mays L*), native to Central and South America, is an important food crop in the world. Whole crop maize silage (WCMS) is the dominating roughage for cows due to its high nutritional value and biological conversion efficiency. With the increasing demand for milk, the domestic yield of WCMS is no longer sufficient for the development of animal husbandry. It is fairly crucial and urgent to introduce new types of roughages. Unconventional feeds are rarely used in formulas or have little understanding of their nutritional properties and feed value (Tian et al. 2020). Previous studies have reported that some unconventional feeds can be treated as animal feed without affecting the health of animals (Zeng et al. 2018; Tian et al. 2020).

*Broussonetia papyrifera* (*BP*), a deciduous tree of the *Moraceae* family, which is mainly distributed over eastern Asia. It has the characteristics of strong adaptability to environment, high nutritional value and good biomass yield (Liao et al. 2014). Straightforward application of *BP* as feed will reduce its nutritional value, because it is rich in anti-nutrient factors including flavonoids and other substances (Han et al. 2016). Ensiling is a practical preservation method for *BP*, which can eliminate most of the anti-nutrient factors (Grant and Ferraretto 2018). Currently, reports have shown that feeding Holstein heifers with *BP* silage (*BPS*) can improve production performance, but the research on the effect of *BPS* on rumen microbes is unavailable. Generally, the structure and types of rumen microbes are closely related to host health (Jami et al. 2013). For example, they have the ability to convert fiber materials in plants into volatile fatty acids (VFAs) and microbial proteins for host. In this study, 16S rRNA high-throughput sequencing was applied to explore the effect of feeding Holstein heifer *BPS* to rumen microbes (Van et al. 2014).

In the present study, effects of replacing WCMS with different proportions of *BPS* on the total tract digestibility, serum biochemical indicators and rumen fermentation parameters of Holstein heifers were investigated. These indicators (total tract digestibility, serum biochemical indicators, and rumen fermentation parameters) were used as markers to explore the function of rumen microbes via Weighted Correlation Network Analysis (WGCNA). This work was carried out to provide a reference for the further application of *BPS* in Holstein heifers, and to alleviate the problem of feed shortage.

## Material and methods

All experimental procedures in this study were approved by the Committee of Animal Experiments of South China Agricultural University (No. 201004152) in accordance with the guidelines for Animal Research outlined in South China Agricultural University.

### Diets, animals and management

*BPS* was obtained from a feed production company (Heyuan, China). The cutting height of hybrid *BP* was between 1.0 and 1.5 m. The main stem and the yellowed leaves were removed, and the 20–30 cm section in front of the branches were maintained. These *BP* samples were cut into 1–2 cm length to make 30–40 kg silage bags, and then sealed at room temperature for 45 days.

The research was conducted in a Yantang Red May cow farm in Yangjiang, China. Twenty Holstein heifers (8 months old) with similar weight (245 ± 24 kg) and genetic background were assigned randomly to four groups of five heifers. All heifers were weighed, marked with numbered identification tags, and inoculated with vaccines (foot-and-mouth disease and epidemic fever) prior to the experiment.

The experimental diets were formulated in accordance with the Chinese feeding standards (China Standard NY/T34, 2004). TMR samples were collected on day 0, 7, 14, 21 and 28. Four dietary treatments containing different proportions of *BPS* (0%, 25%, 50% and 75%) as substitute for WCMS (labelled T0, T25, T50 and T75, respectively) were tested in Holstein heifers. The dry matter (DM), crude protein (CP), ether extract (EE), acid detergent fiber (ADF) and neutral detergent fiber (NDF) were measured by the procedures of the Association of Official Analytical Chemists (AOAC) (AOAC 2000). The nutrient composition of *BPS* and WCMS, the dietary ingredients and the nutrient compositions for this trial are in accordance with Tian (2020). The heifers were fed at 8:00 and 18:00, and water was provided ad libitum throughout the experiment. The feeding adaption period was 7 days, and the experiment period was 30 days.

### Total tract digestibility

During the 27–30 days of the trial, after morning feeding at 0 h, 4 h, 8 h, and 12 h, fecal was collected by the rectum fecal method (Huang et al. 2020). Fecal samples from an individual Holstein heifer are pooled together during this time. Acid insoluble ash (AIA) was calculated as an internal marker to compute the total tract digestibility (Huang et al. 2019). The nutrient digestibility of diets were calculated using the following equation:

$$ {\text{Nutrient digestibility }}\left( \% \right) \, = { 1}00 \, \times \, [{1 - }({\text{AIA diet}}/{\text{AIA feces}}) \, \times \, \left( {{\text{nutrient feces}}/{\text{nutrient diet}}} \right)] $$

### Serum biochemical indicators

On day 30, blood samples were collected via the jugular vein 4 h after the morning feeding using tubes (BD Vacutainer, BD and Co., Franklin Lakes, NJ), and then left for 30 min and centrifuged at 4000 r/min for 15 min. Serum biochemical indicators including aspartate aminotransferase (AST), alanine aminotransferase (ALT), urea (UREA), lactic dehydrogenase (LDH), alkaline phosphatase (ALP), total protein (TP), albumin (ALB), and total cholesterol (TC) were determined by using a biochemical auto-analyzer (Hitachi automatic biochemical analyzer 7080, Tokyo, Japan). Triiodothyronine (T3) and tetraiodothyronine (T4) were detected by DFM-96 r-radio immune counter.

### Rumen fermentation parameters

On the last day of the experiment, the rumen fluid samples (250 mL) were collected via rumen cannula 4 h after the morning feeding (Shen et al. 2012). The rumen fluid samples were filtered with four layers of gauze, which had undergone high-pressure sterilisation. The filtered rumen fluid was separated into two 50 mL centrifuge tubes and three 2 mL cryogenic vials. The cryogenic vial was immediately frozen in liquid nitrogen and subsequently stored in a freezer maintained at − 80 °C to determine the rumen microbe. The 50 mL centrifuge tubes were collected for the determination of pH value, ammonia nitrogen (NH_3_-N) and VFAs. VFAs such as acetic acid (AA), propionic acid (PA), isobutyric acid (IBA), isovaleric acid (IVA), valeric acid (VA) and butyric acid (BA) were analysed by high-performance liquid chromatography (HPLC). The concentration of NH_3_-N was determined according to the previous study (Broderick and Kang 1980). We calculated the ratio of the molar concentration of AA to the molar concentration of PA (AA/PA).

### Rumen microbes

Microbial DNA was extracted from rumen fluid via bacterial DNA isolation kit (Omega Bio-Tek, Norcross, GA, USA), and primers 338F (5ʹ-ACTCCTACGGGAGGCAGCAG-3ʹ) and 806R (5ʹ-GGACTACHVGGGTWTCTAAT-3ʹ) were used to amplify the V3-V4 region of the 16S rRNA gene. High-throughput sequencing was performed using the Illumina MiSeq platform (San Diego, CA, USA). All sequences with low-quality scores and sequences in the overlap region were discarded. The fastQ file was analysed using the downstream computing pipeline of QIIME (Caporaso et al. 2010) and the default minimum quality threshold was 25. Aggregating sequences at a sequence similarity level of 97% (Edgar et al. 2010), the RDP classifier assigned representative Operational Taxonomic Unit (OTU) to bacterial classifications with a confidence threshold of 0.8.

#### Alpha and beta diversity analysis

*Methanococcus* *jannaschii* (L77117) was used in the outer group to root the phylogenetic tree. Subsequently, the OTU table was generated from QIIME and assembled into Phyloseq objects together with the mapping file. Alpha diversity was calculated using standard diversity metrics accessed by Phyloseq. The Principal Co-ordinates Analysis (PCoA) was conducted based on the Unweighted Unifrac distances. Adonis multivariate analysis of variance (Adonis) was calculated to test the difference in Beta diversity among groups. LDA Effect Size (LEfSe) analysis is regarded as an analytical tool for discovering and interpreting high-dimensional data biometric identifiers, which can be used to find biometric identifiers with statistical differences between groups.

### Statistical analysis

Apparent digestibility, serum biochemical indicatores and rumen fermentation were analysed using SAS 9.4 (SAS Inst Inc, Cary, NC, USA). Briefy, INFLUENCE statement was used to eliminate outliers, GLM procedure was invoked for data processing, LSMEANS statement calculated the least square mean of processing and Tukey method was used for multiple comparison. The model used for data processing was *Y*_*ij*_ = *μ* + *Τ*_*i*_ + *ε*_*ij*_, where *Y*_*ij*_ is the dependent variable value of different treatment groups, *μ* is the general mean, *T*_*i*_ is the fixed effect of treatment, and *ε*_*ij*_ is the random error. Orthogonal polynomial contrasts were used to analyse the effects of the different BPS inclusion levels on the apparent digestibility, serum biochemical indicators and rumen fermentation. The experimental data were expressed in tables with mean value and standard error of mean (SEM), the significant difference was accepted at *P* < 0.05.

WGCNA was adopted as a module for correlation analysis. WGCNA is a system biology method used to describe the pattern of factor associations between different samples. It can identify synergistically changing factor sets, and identify biomarkers based on the inter-connectivity of the factor sets and the correlation between the factor sets and the phenotype. The numbers at the top of the matrix represent correlations, and the numbers below represent *p*-values. The scale colors denote whether the correlation is positive (closer to 1, red squares) or negative (closer to  −1, green squares) between the bacteria and the efficiency parameters.

## Results

### Total tract digestibility

The total tract digestibility is shown in Table 1. We found that the digestibility of NDF, ADF, and CP increased linearly (*P* < 0.05) with the increase in *BPS*. T75 possessed higher digestibility of NDF (61.16% vs. 46.71%) and ADF (53.17% vs. 37.24%) than T0. The CP digestibility was higher in T75 (59.80%) and T50 (56.48%) than in T0 (40.25%).

**Table 1:** Effects of *BPS* on apparent digestibility of Holstein heifers

Item	Dietary treatment				SEM	Contrast	
	T0	T25	T50	T75		Line	Quad
Total tract digestibility, %							
NDF	46.71	52.11	52.97	61.16	3.19	0.006	0.668
ADF	37.24	40.43	41.94	53.17	4.74	0.028	0.413
CP	40.25	50.22	56.48	59.80	5.43	0.016	0.553
EE	43.72	67.20	49.72	56.35	7.07	0.527	0.250

T0, 0% *BPS*; T25, 25% *BPS*; T50, 50% *BPS*; T75, 75% *BPS*; SEM, standard error of the mean. Line: linear; quad: quadratic

Means within the same row without the same letter superscripts are significantly

### Serum biochemical indicators

Table 2 reports the effect of dietary treatments on serum biochemical indicators. Our results showed no significant differences in the strengths of AST, ALT, TC, ALP, T3 and T4 among groups (*P* > 0.05). The concentrations of UREA (*P* = 0.057) and LDH (*P* = 0.088) tended to increase linearly with the increase in *BPS*. T50 and T25 had higher concentrations of TP (54.63% vs. 58.38% vs. 35.20%) and ALB (26.08% vs. 27.70% vs. 18.53%) than T0. The strengths of TP and ALB increased quadratically (*P* < 0.05) with the increase in *BPS* in the diet.

**Table 2:** Effects of *BPS* on serum biochemical indicatores of Holstein heifers

Item	Dietary treatment				SEM	Contrast	
	T0	T25	T50	T75		Line	Quad
AST, (U/L)	37.68	52.88	48.78	48.15	2.80	0.277	0.166
ALT, (U/L)	15.00	20.30	21.50	17.13	1.28	0.504	0.073
UREA, (mmol/L)	3.44	4.84	4.72	4.82	0.24	0.057	0.151
LDH, (U/L)	655.50	891.50	893.50	895.50	47.09	0.088	0.203
ALP, (U/L)	83.48	92.33	81.65	88.13	5.87	0.956	0.928
TP, (g/L)	35.20	58.38	54.63	47.40	3.22	0.174	0.011
ALB, (g/L)	18.53	27.70	26.08	22.85	1.30	0.245	0.011
TC, (mmol/L)	1.44	1.98	1.91	1.70	0.13	0.558	0.171
T3, (nmol/L)	2.19	2.18	2.36	2.31	0.09	0.539	0.908
T4, (nmol/L)	201.18	200.03	208.25	194.98	6.87	0.881	0.695

T0, 0% *BPS*; T25, 25% *BPS*; T50, 50% *BPS*; T75, 75% *BPS*; SEM, standard error of the mean. Line: linear; quad: quadratic; AST: glutamic oxalacetic transaminase; ALT: alanine aminotransferase; Urea: UREA; LDH: lactic dehydrogenase; ALP: alkaline phosphatase; TP: total protein; ALB: albumin; TC: total cholesterol; T3: triiodogenic thyrogen; T4: tetraiodothyroxine

Means within the same row without the same letter superscripts are significantly

### Rumen fermentation parameters

The rumen fermentation parameters are presented in Table 3. The substitution of *BPS* in the diet for WCMS did not alter the pH value and the concentrations of AA, PA, IBA, BA and VA of rumen. As the proportion of BPS in the diet increased, the density of NH_3_-N decreased linearly (*P* < 0.05). The NH_3_-N concentration was lower in T75 (3.22 mg/dL) than in T0 (5.87 mg/dL) group. Moreover, we found that the ration of AA/PA (*P* = 0.089), and the deepness of IVA (*P* = 0.084) have a linear increase trend with the addition of *BPS*.

**Table 3:** Effects of *BPS* on rumen fermentation of Holstein heifers

Items	Dietary treatment				SEM	Contrast	
	T0	T25	T50	T75		Line	Quad
pH	6.93	7.00	7.00	7.05	0.12	0.482	0.935
NH_3_-N (mg/dL)	5.87	5.56	3.74	3.22	0.50	0.024	0.911
VFA (mmol/L)							
AA	33.07	33.89	31.66	32.55	1.79	0.826	0.993
PA	8.59	9.12	8.93	8.91	0.50	0.868	0.809
IBA	0.35	0.41	0.37	0.37	0.02	0.648	0.964
BA	4.23	4.55	3.89	3.86	0.24	0.447	0.741
IVA	0.59	0.69	0.47	0.44	0.04	0.084	0.819
VA	0.27	0.29	0.27	0.24	0.02	0.458	0.536
AA/PA	3.86	3.72	3.57	3.67	0.05	0.072	0.158

T0, 0% *BPS*; T25, 25% *BPS*; T50, 50% *BPS*; T75, 75% *BPS*; SEM, standard error of the mean. Line: linear; quad: quadratic; pH: pH value; NH_3_-N: ammonia nitrogen; VFA: volatile fatty acids; AA: acetic acid; PA: propionic acid; IBA: isobutyric acid; BA: butyric acid; IVA: isovaleric acid; VA: valeric acid; AA/PA: acetic acid/propionic acid

Means within the same row without the same letter superscripts are significantly

### Sequencing depth, coverage, alpha and beta diversity

**Fig. 1:**
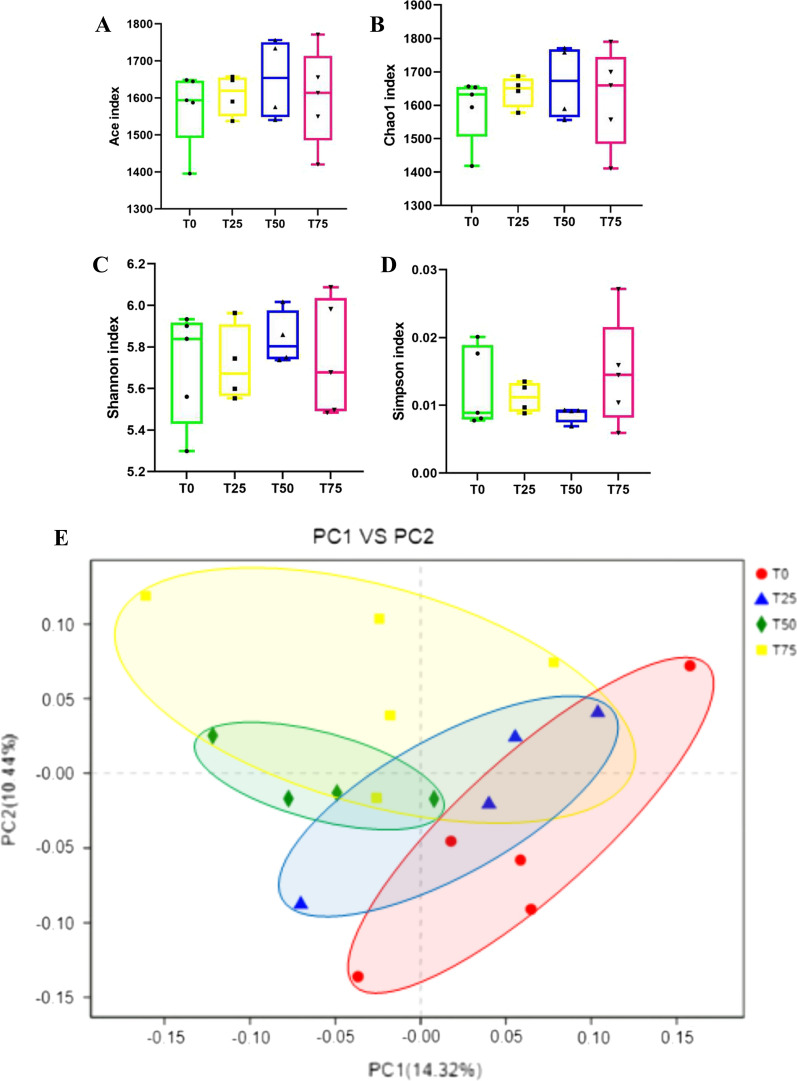
Rumen microbiota diversity. **A** Chao1 index. **B** Ace index. **C** Simpson index. **D** Shannon index. **E** The Principal Co-ordinates Analysis (PCoA)

The V3-V4 regions of the 16S rRNA gene were sequenced in 18 samples. After quality control and chimera removal, a total of 352,476 high-quality sequences were retained, with an average of 19,582 sequences per sample. The mean length of these sequences was 421-460 bp. According to the 97% similarity, OTU clustering was performed, and a total of 2016 effective OTU were obtained. In our research, we calculated the richness indices (Ace and Chao1) and diversity indices (Simpson and Shannon) to measure the change in rumen microbe. *BPS* had no effect on these indices (Fig. 1A–D). In our study, PCoA and Adonis analysis were calculated to measure the Beta diversity of rumen microbe among groups. Combining Fig. 1E and Table 4, it could be seen that adding *BPS* to the diet did not change the Beta diversity of rumen microbe.

**Table 4:** Adonis multivariate analysis of variance analysis

Items	R.^2^	P
T0-T25	0.1100	0.552
T0-T50	0.1612	0.094
T0-T75	0.1463	0.142
T25-T50	0.1294	0.484
T25-T75	0.1347	0.306
T50-T75	0.1406	0.254

T0, 0% *BPS*; T25, 25% *BPS*; T50, 50% *BPS*; T75, 75% *BPS*

### Change in rumen bacterial community

**Fig. 2:**
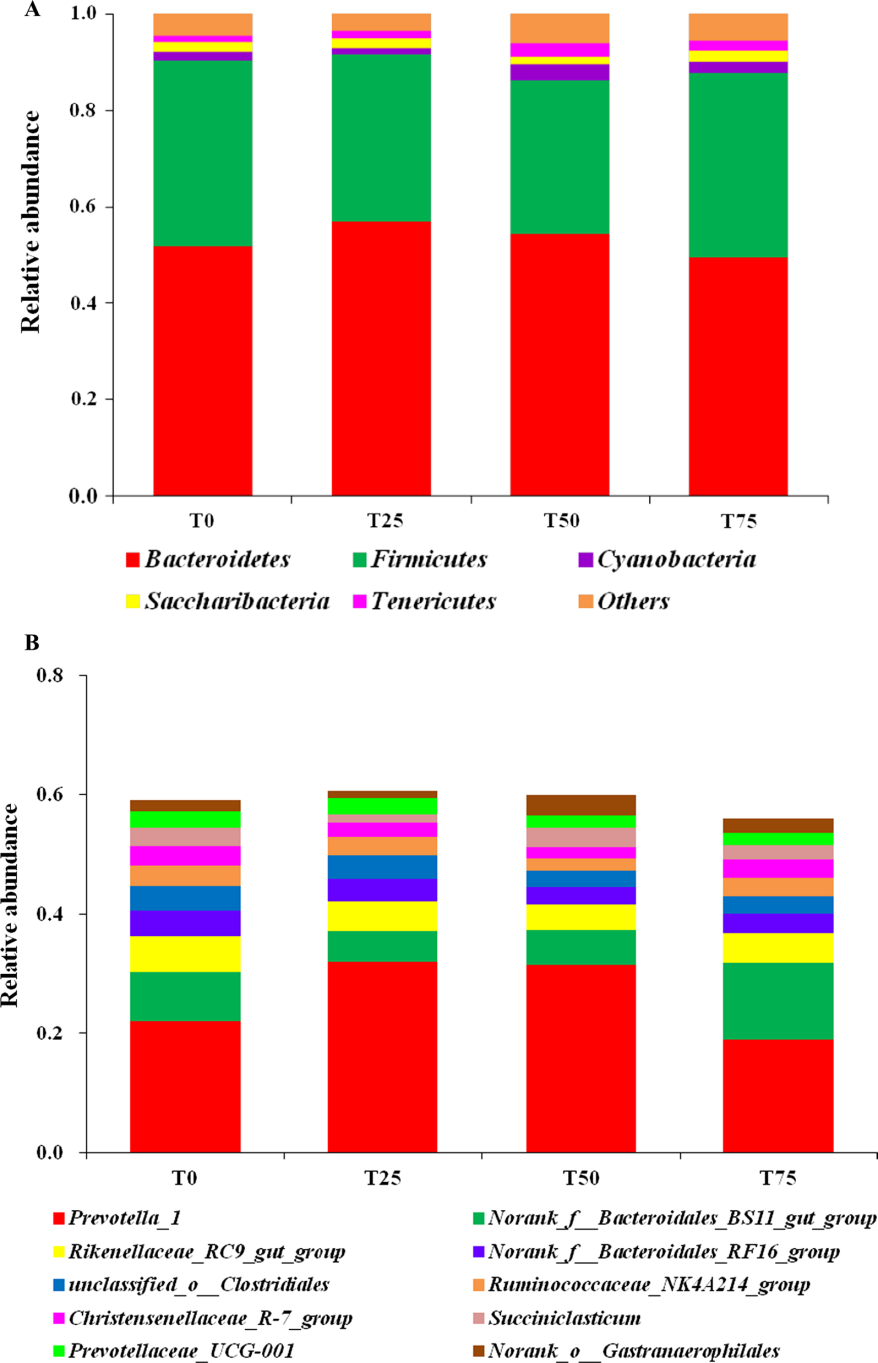
Effect of *B. papyrifera* silage on rumen microbiota composition. The rumen micobiota composition of Holstein heifers in T0, T25, T50 and T75 group at **A** phylum and **B** genus level

**Fig. 3:**
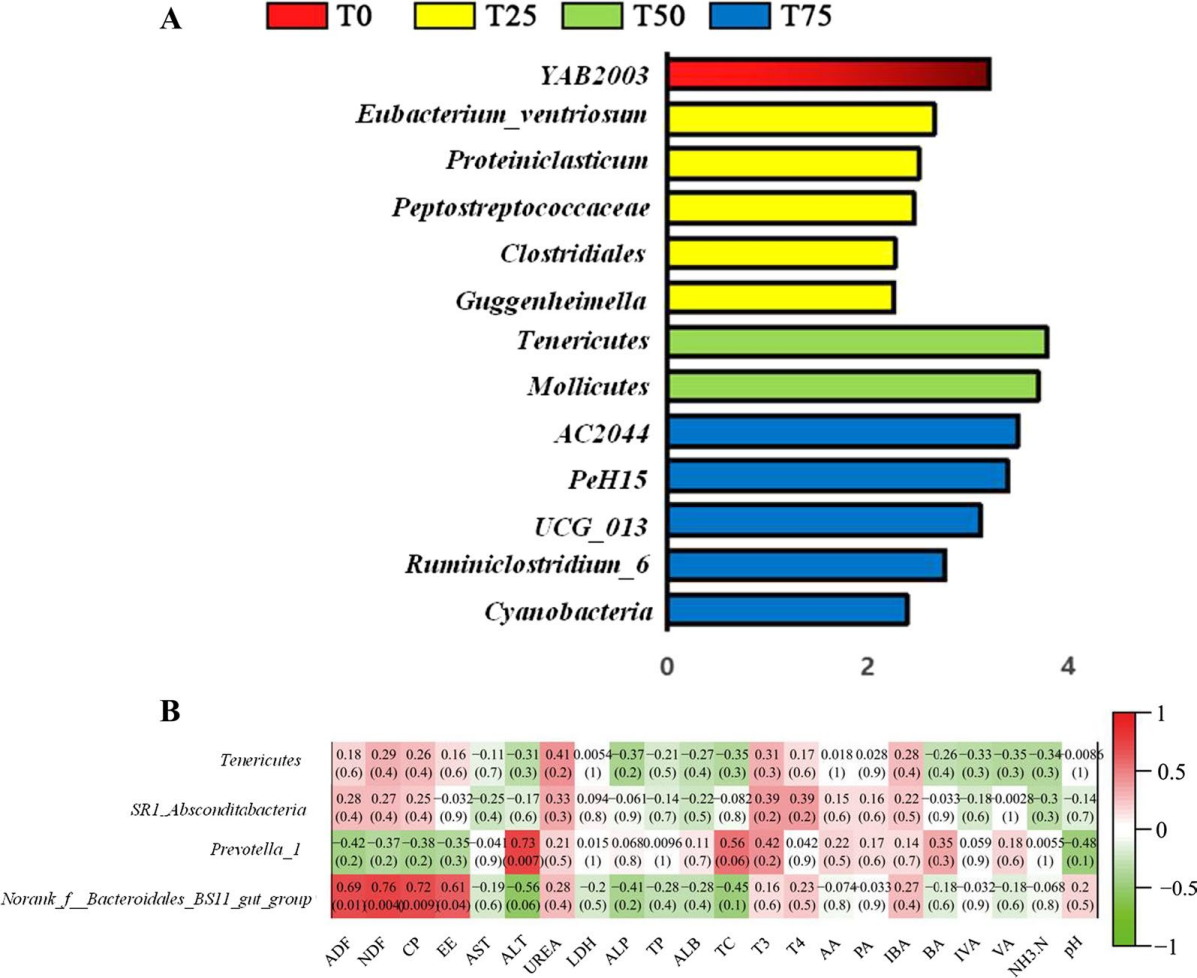
The LDA Effect Size analysis (LEfSe) **A** of Holstein heifers in T0, T25, T50 and T75 group. **B** Correlation between the statistically different bacteria at the genus and digestibility/rumen fermentation parameters/serum biochemical indicators

In our study, we selected bacteria with the top 10 relative abundances at the phylum and genus level for further analysis. In term of phylum level, the predominant bacteria were *Bacteroidetes* (51.79%, 56.85%, 54.35% and 49.4% on average), *Firmicutes* (38.44%, 34.82%, 31.87% and 38.38% on average), and *Cyanobacteria* (1.92%, 1.21%, 3.36% and 2.30% on average) in T0, T25, T50 and T75 (Fig. 2A and Additional file 1: Table S1). At the genus level, the dominant bacteria were *Prevotella_1* (22.10%, 32.04%, 31.65% and 18.94% on average), *Norank_f__Bacteroidales_BS11_gut_group* (8.38%, 5.15%, 5.79% and 12.93% on average) and *Rikenellaceae_RC9_gut_group* (5.88%, 5.05%, 4.22% and 4.98% on average) in T0, T25, T50 and T75 (Fig. 2B and Additional file 2: Table S2). In general, diet did not alter the order of rumen dominant bacteria. However, the relative abundances of *Tenericutes* and *SR1–Absconditabacteria* increased linearly (*P* < 0.05), and *Norank_f__Bacteroidales_BS11_gut_group* increased quadratically (*P* < 0.05) with the addition of *BPS* (*P* < 0.05). As shown in Fig. 3A, 13 different bacteria were identified including 1 phylum (*Tenericutes*), 1 class (*Mollicutes*), 1 family (*Clostridiales*), and 10 genera (*YAB2003*, *Eubacterium__ventriosum*, *Proteiniclasticum*, *Guggenheimella*, *Peptostreptococcaceae*, *Cyanobacteria*, *UCG_013*, *Ruminiclostridium_6*, *PeH15* and *AC2044*).

### Correlation analysis

In the present study, we selected bacteria that are statistically different at the phylum and the genus level for correlation analysis with total tract digestibility/rumen fermentation parameters/serum biochemical indicators and the results are shown in Fig. 3B. ALT concentration was positively correlated with the abundance of *Prevotella-1* (r = 0.73; *P* = 0.007). The relative abundance of *Norank_f__Bacteroidales_BS11_gut_group* correlated positively with the digestibility of ADF, NDF, CP and EE (r = 0.69, *P* = 0.01; r = 0.76, *P* = 0.004; r = 0.72, *P* = 0.009; r = 0.61, *P* = 0.04, respectively), while tend to correlate negatively with the concentration of ALT (r = − 0.56, *P* = 0.06).

### Function prediction

**Fig. 4:**
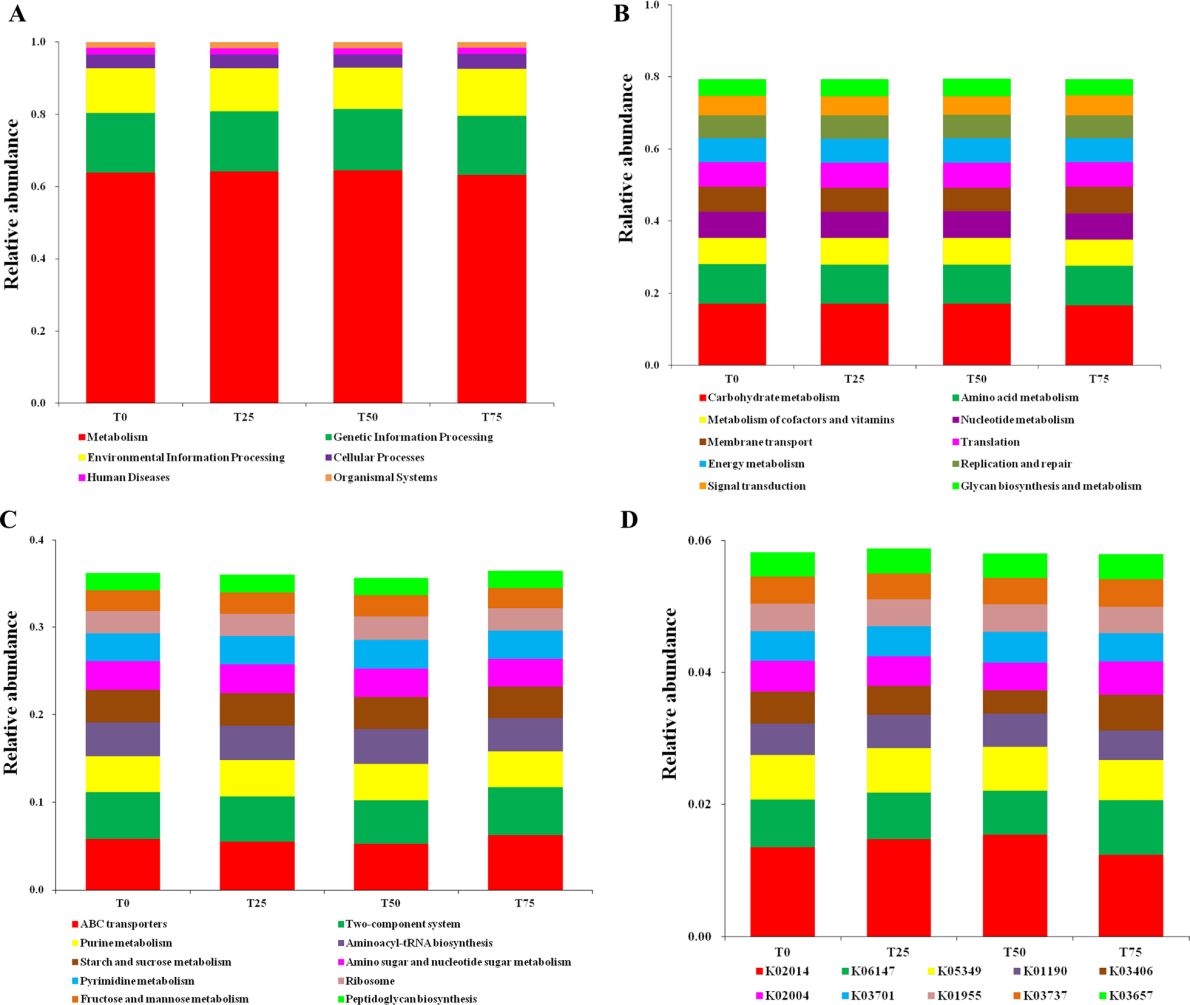
Function prediction on rumen microbes of the four treatments. **A** First level; **B** Second level; **C** Third level; **D** KEGG orthologues level

The functional prediction of rumen microbes were performed by Tax4Fun. At first level, the pathways were focused on metabolism, genetic information processing, environmental information processing, cellular processes, human diseases and organismal systems (Fig. 4A). The top ten pathways at second level were carbohydrate metabolism, amino acid metabolism, metabolism of cofactors and vitamins, nucleotide metabolism, membrane transport, translation, energy metabolism, replication and repair, signal transduction and glycan biosynthesis and metabolism (Fig. 4B). ABC transporters, two component system, purine metabolism, aminoacyl-tRNA biosynthesis, starch and sucrose metabolism, amino sugar and nucleotide sugar metabolism, pyrimidine metabolism, ribosome, fructose and mannose metabolism and peptidoglycan biosynthesis were the top ten pathways at third level (Fig. 4C). At level K, the top ten KEGG orthologues were K02014, K06147, K05349, K01190, K03406, K02004, K03701, K01955, K03737 and K03657 (Fig. 4D).

## Discussion

In the present study, compared with T0, Holstein heifers fed with *BPS* had higher nutrient utilisation, which is consistent with the findings of Tao et al. (2020), that is, adding 15% *BPS* to the diet can increase the body weight and feed digestibility of cattles. The higher nutrient digestibility in heifers might be explained by flavonoids and alkaloids in *BPS* (Chen et al. 2020). Alkaloids are synthesised from different amino acids or their direct derivatives, and they are generally related to important physiological activities. A previous study has also reported that these bioactive substances can promote the production of intestinal mucus (Chen et al. 2020). In addition, the results of 16 s high-throughput sequencing in the present study suggested that the relative abundance of *Prevotella-1* (which is widely involved in the degradation of starch and protein in the rumen) tends to increase quadratically as the *BPS* increased. And Li et al. (2019) have reported that some bioactive substances can increase the number of rumen microbes, thereby increasing the digestibility of feed. However, nutrient digestibility is a complex trait affected by multiple parameters. The gradual increase in nutrient digestibility may only reflect the better digestibility of *BPS*. The mechanism by which *BPS* improves the nutrient digestibility of heifers needs further investigation.

Serum biochemical indicators reveal the utilisation of nutrients in the body. TC is the main indicator that reflects the body's metabolic energy balance and lipid metabolism (Maan et al. 2018). AST, ALT and LDH are used to assess whether the liver's ability to metabolise proteins and amino acids is abnormal (Jiang et al. 2014). T3 and T4 are the main secretions of the thyroid gland and can increase the oxygen consumption rate of most tissues (Hong et al. 2018). ALP is an important functional enzyme in the process of bone formation and bone turnover. As we can see from the Table 2, feeding *BPS* to heifers in current study did not change the level of these biochemical indicators. Serum TP and ALB reflect the absorption and hydrolysis of dietary protein in the body (Tang et al. 2005). When compared with T0, *BPS* groups possessed higher TP and ALB concentrations; these findings might be related to higher CP digestibility found in *BPS* groups. From the above results, we repute that heifers fed with *BPS* have higher nutrient digestibility, and correspondingly increase the level of relevant biochemical indicators. Adding *BPS* to the diet will not harm the normal physiological activities of heifers.

NH_3_-N content reflects the degradation rate of CP in feed and the dynamic balance of microbial protein synthesis. Generally, its concentration is positively correlated with the dietary CP level. Interestingly, in our research, Holstein heifers fed with high-level *BPS* possessed a higher CP digestibility, but the rumen NH_3_-N concentration was significantly reduced. *BPS* is rich in many active substances, which may change the abundance of some crucial bacteria, thereby promoting the body's utilisation of NH_3_-N. The digestion of microbe occupies a very important position in ruminant nutrition; it is also the fundamental reason why ruminant can survive almost entirely on roughage. The diet addition of *BPS* in this research altered the relative abundance of various bacteria (*Prevotella-1, Tenericutes*, and *YAB2003*) associated with rumen fermentation (Purushe et al. 2010; Zou et al. 2019). However, the response of the microbe is complex and dynamic, and the regulatory mechanisms are rarely discovered. In general, feeding heifers with *BPS* reduced the level of NH_3_-N, did not affect other rumen fermentation parameters, and enhanced rumen function.

In line with the previous study (Jiang et al. 2021), our results found that the rumen dominant bacteria phyla were *Bacteroidetes* and *Firmicutes*, regardless of dietary treatments. However, the third dominant bacterium in this study was *Cyanobacteria*, rather than *Proteobacteria,* as reported in many studies. *Cyanobacteria* is a type of gram-negative bacteria. Mao et al. (2012) reported that a variety of *Cyanobacteria* possesses a significant negative correlation with the concentration of rumen AA, PA and BA. This difference may be due to factors, such as regions, environment, diets, and management mode (Henderson et al. 2016). The main core genus in the present study is *Prevotella-1,* which is consistent with the rumen dominant genus reported by most scholars (Avguštin et al. 2001; Castillo-Lopez et al. 2018). *Prevotella-1* has diverse functions, such as extensive involvement in the degradation of starch, proteins, and peptides in the rumen (Miyazaki et al. 1997; Wallace et al. 1997; Purushe et al. 2010), as well as maintaining glucose homeostasis and passing through host of gluconeogenesis that participates in PA fermentation (Purushe et al. 2010). Our results suggested that the gradual addition of BPS in diet tended to increase quadratically the relative abundance of *Prevotella-1*, indicating that an appropriate replacement amount of *BPS* can improve the growth performance of Holstein heifers.

At the phylum level, the relative abundances of *Tenericutes* and *SR1–Absconditabacteria* increased linearly with the increase in *BPS. Tenericutes* is regarded as hemicellulose-degrading bacteria, with AA and BA as the main products (Zou et al. 2019); it has the potential to regulate host metabolism (Terova et al. 2021). In this experiment, the relative abundance of *Tenericutes* in the T50 was significantly higher than that in the T0, but did not possess higher AA and BA concentrations. These differences may be due to some low relative abundances but the key bacteria affect the concentrations of AA and BA. *SR1-Absconditabacteria* is one of the common bacteria in the ruminant rumen (Liu et al. 2019; Li et al. 2021). However, studies on this type of bacteria are limited, and its function remains unfamiliar. At the genus level, the relative abundance of *Norank_f__Bacteroidales_BS11_gut_group* increased quadratically with the increase in *BPS*. *Norank_f__Bacteroidales_BS11_gut_group* is one of the main degraders of many complex polysaccharides in plant cell walls and hemicellulose monomer sugar fermentation (Ren et al. 2020). Liu et al. (2017) found that feeding starter feeds to weaned lambs can increase the relative abundance of the *Norank_f__Bacteroidales_BS11_gut_group*. Moreover, cows fed with monensin exhibit similar effect (Scharen et al. 2017). In general, starter feed or monensin can improve the structure and diversity of rumen bacteria. The relative abundance of *Norank_f__Bacteroidales_BS11_gut_group* may serve as a biomarker of rumen microbial structure, and the microbial structure of heifers fed with *BPS* becomes more complete.

LEfSe analysis can identify biomarkers with statistical differences between groups. Our results suggested that *YAB2003* was significantly over-represented in the T0. It is considered as saponin functional bacteria and can cooperate with saponin to improve rumen fermentation and milk production performance of cows. In addition, several bacteria related to body metabolism, such as sugar metabolism (*Lachnospiraceae_AC2044_group*) and immunomodulation (*Ruminococcaceae_UCG-013*) (Zhang et al. 2017) were found in the *BPS* groups. This difference is due to *BPS*, which is rich in a variety of active substances, thereby increasing the relative abundance of some bacteria related to body metabolism. Studies on *BPS* as a feed for ruminants to regulate the rumen microbial community are few, and the mechanism of its regulation on the rumen microbes needs to be further explored.

Ruminant rumen microbes are responsible for degrading and fermenting different types of feeds to provide a large number of nutrients for the growth, reproduction, maintenance and lactation of the host. We found that the statistically different bacteria were mainly associated with serum biochemical indicators, that is, the ALT concentration is significantly positively correlated with the abundance of *Prevotella-1*. However, *Prevotella-1* has always been regarded as cellulolytic bacteria (Purushe et al. 2010). The functions of microbes are diverse, and our understanding of rumen microbes is insufficient. Moreover, a previous study suggested that bacteria with an abundance of < 0.1% can play an important role (Morgavi et al. 2013), but these bacteria are often overlooked due to technical restrictions. More massive datasets that include more variables should be used, and more sophisticated methodologies are needed to unravel further these interrelations (Wallace et al. 2017).

In conclusion, this study revealed that the effect of using *BPS* as a substitute for WCMS on rumen microbes, total tract digestibility, rumen fermentation parameters, and serum biochemical indicators of Holstein heifers. Holstein heifers fed with *BPS* did not change the diversity and uniformity of rumen microbes. In addition, we select bacteria with statistically different in this study to carry out correlation analysis with the above indicators (total tract digestibility, rumen fermentation parameters, and serum biochemical indicators), aiming to explore their potential functions. For example, *Prevotella-1* may not only be considered as a cellulolytic bacteria, it may also be related to serum biochemical indicators. In the whole, this research provides a further basis for the application of *BPS* in heifers, and also reveals some potential functions of bacteria.

## Supplementary Information

**Additional file 1: Table S1.** Effects of *BPS* on rumen bacteria (phylum-level) of Holstein heifers.

**Additional file 2: Table S2.** Effects of *BPS* on rumen bacteria (genus-level) of Holstein heifers.

## Data Availability

The sequences in this study were submitted to the Sequence Read Archive (SRA) and a BioProject number PRJNA816335 was obtained.
