# Supplementary material for: Effects of Broussonetia papyrifera silage on rumen fermentation parameters and microbes of Holstein heifers
Source: AMB Express. 2022 May 25;12:62. doi: 10.1186/s13568-022-01405-x (PMC9133286; doi:10.1186/s13568-022-01405-x)
Supplement: Supplementary file 1 — Additional file 1: Table S1. Effects of BPS on rumen bacteria (phylum-level) of Holstein heifers. [file 13568_2022_1405_MOESM1_ESM.docx]

Table S1 Effects of *BPS* on rumen bacteria (phylum-level) of Holstein heifers

| Species name | Dietary treatment | | | | SEM | *Contrast* | |
| --- | --- | --- | --- | --- | --- | --- | --- |
|  | T0 | T25 | T50 | T75 |  | line | quad |
| *Bacteroidetes* | 51.79 | 56.85 | 54.35 | 49.4 | 2.14 | 0.624 | 0.286 |
| *Firmicutes* | 38.44 | 34.82 | 31.87 | 38.38 | 2.33 | 0.884 | 0.325 |
| *Cyanobacteria* | 1.92 | 1.21 | 3.36 | 2.30 | 0.33 | 0.228 | 0.779 |
| *Saccharibacteria* | 2.01 | 2.05 | 1.53 | 2.31 | 0.17 | 0.805 | 0.310 |
| *Tenericutes* | 1.38 | 1.52 | 2.67 | 2.07 | 0.2 | 0.047 | 0.299 |
| *Proteobacteria* | 1.22 | 0.96 | 1.26 | 1.15 | 0.11 | 0.924 | 0.767 |
| *Fibrobacteres* | 1 | 0.49 | 1.45 | 0.68 | 0.21 | 0.997 | 0.767 |
| *SR1_--_Absconditabacteria* | 0.64 | 0.49 | 1.01 | 1.33 | 0.11 | 0.004 | 0.208 |
| *Spirochaetaes* | 0.6 | 0.52 | 0.97 | 0.91 | 0.14 | 0.305 | 0.964 |
| *Verrucomicrobia* | 0.41 | 0.62 | 0.79 | 0.78 | 0.11 | 0.224 | 0.646 |

Abbreviation: T0, 0% *BPS*; T25, 25% *BPS*; T50, 50% *BPS*; T75, 75% *BPS*; SEM, standard error of the mean. line, linear; quad, quadratic.
