# Supplementary material for: Effects of Broussonetia papyrifera silage on rumen fermentation parameters and microbes of Holstein heifers
Source: AMB Express. 2022 May 25;12:62. doi: 10.1186/s13568-022-01405-x (PMC9133286; doi:10.1186/s13568-022-01405-x)
Supplement: Supplementary file 2 — Additional file 2: Table S2. Effects of BPS on rumen bacteria (genus-level) of Holstein heifers. [file 13568_2022_1405_MOESM2_ESM.docx]

Table S2 Effects of *BPS* on rumen bacteria (genus-level) of Holstein heifers

| Species name | Dietary treatment | | | | SEM | *Contrast* | |
| --- | --- | --- | --- | --- | --- | --- | --- |
|  | T0 | T25 | T50 | T75 |  | line | quad |
| *Prevotella_1* | 22.10 | 32.04 | 31.65 | 18.94 | 2.79 | 0.673 | 0.053 |
| *Norank_f__Bacteroidales_BS11_gut_group* | 8.38 | 5.15 | 5.79 | 12.93 | 1.24 | 0.148 | 0.033 |
| *Rikenellaceae_RC9_gut_group* | 5.88 | 5.05 | 4.22 | 4.98 | 0.40 | 0.330 | 0.347 |
| *Norank_f__Bacteroidales_RF16_group* | 4.29 | 3.78 | 2.89 | 3.35 | 0.61 | 0.521 | 0.716 |
| *Unclassified_o__Clostridiales* | 4.06 | 3.81 | 2.82 | 2.84 | 0.50 | 0.322 | 0.901 |
| *Ruminococcaceae_NK4A214_group* | 3.43 | 3.09 | 1.98 | 3.07 | 0.34 | 0.474 | 0.318 |
| *Christensenellaceae_R-7_group* | 3.33 | 2.51 | 1.92 | 3.03 | 0.36 | 0.650 | 0.216 |
| *Succiniclasticum* | 2.97 | 1.35 | 3.21 | 2.47 | 0.40 | 0.917 | 0.591 |
| *Prevotellaceae_UCG-001* | 2.78 | 2.71 | 2.16 | 2.09 | 0.25 | 0.269 | 0.995 |
| *Norank_o__Gastranaerophilales* | 1.92 | 1.20 | 3.34 | 2.26 | 0.33 | 0.240 | 0.769 |

Abbreviation: T0, 0% BPS; T25, 25% BPS; T50, 50% BPS; T75, 75% BPS; SEM, standard error of the mean. line, linear; quad, quadratic.
